# lpa1-5525: A New lpa1 Mutant Isolated in a Mutagenized Population by a Novel Non-Disrupting Screening Method

**DOI:** 10.3390/plants8070209

**Published:** 2019-07-06

**Authors:** Giulia Borlini, Cesare Rovera, Michela Landoni, Elena Cassani, Roberto Pilu

**Affiliations:** 1 Department of Agricultural and Environmental Sciences-Production, Landscape, Agroenergy-Università degli Studi di Milano, Via Celoria 2, 20133 Milan, Italy; 2 Department of Biosciences-Università degli Studi di Milano, Via Celoria 26, 20133 Milan, Italy

**Keywords:** maize, low phytic acid, regional mutagenesis, *Ac* transposon, density assay, free phosphate, Chen’s assay, PCR based molecular marker

## Abstract

Phytic acid, or myo-inositol 1,2,3,4,5,6-hexakisphosphate, is the main storage form of phosphorus in plants. It is localized in seeds, deposited as mixed salts of mineral cations in protein storage vacuoles; during germination, it is hydrolyzed by phytases to make available P together with all the other cations needed for seed germination. When seeds are used as food or feed, phytic acid and the bound cations are poorly bioavailable for human and monogastric livestock due to their lack of phytase activity. Therefore, reducing the amount of phytic acid is one strategy in breeding programs aimed to improve the nutritional properties of major crops. In this work, we present data on the isolation of a new maize (*Zea mays* L.) low phytic acid 1 (lpa1) mutant allele obtained by transposon tagging mutagenesis with the *Ac* element. We describe the generation of the mutagenized population and the screening to isolate new lpa1 mutants. In particular, we developed a fast, cheap and non-disrupting screening method based on the different density of lpa1 seed compared to the wild type. This assay allowed the isolation of the lpa1-5525 mutant characterized by a new mutation in the *lpa1* locus associated with a lower amount of phytic phosphorus in the seeds in comparison with the wild type.

## 1. Introduction

Phytic acid (*myo*-inositol-1,2,3,4,5,6-hexakisphosphate, or InsP6) is the most common form of phosphate present in cereal kernels as well as in seeds of most plants [1,2]. Cereals’ seeds contain on average 10 mg of phytic acid, which account from 65% to 90% of the total phosphorus present inside the seed [3]. Among cereals, maize possesses a great importance not only in human and animal nutrition but also in the use of its derivatives in many industrial sectors. As animal feed, maize can be considered one of the most important staple foods available and, in many cases, is indispensable for the formulation of animal rations. Moreover, maize is a model plant in genetic studies aimed at understanding the role of genes involved in biosynthetic pathways and in plant morphogenesis. For those reasons, maize is considered one of the most important plants studied and used in genetic improvement programs.

Phytic acid is synthesized in the endoplasmic reticulum, and then is deposited in protein bodies organized in specific structures called globoids as a mixture of phytic salts of several cations, such as potassium, iron, zinc and magnesium [4]. In cereals’ seeds, the phytates are mainly localized in the embryo (80%) and in the aleurone layer (20%) [1]. During germination, the phytate salts are broken down by phytase activity releasing free phosphate, minerals and myo-inositol, necessary for seedling growth [5]. Furthermore, phytic acid, by firmly chelating iron cations, is able to counteract the formation of reactive oxygen species and is thus involved in the preservation of viability of plant seeds [6,7]. Phytic acid is poorly digested by monogastric animals, and since it is a chelator of cations, it is considered an anti-nutritional factor. Moreover, as it is not assimilated, it is expelled with manure, becoming a pollutant of cultivated land and contributing to the eutrophication of surface waters [8]. It has been estimated that nearly 50% of elemental P used in global agricultural activities is accumulated in the phytic acid fraction. For these reasons, its reduction or elimination in the seeds is a major challenge in genetic improvement programs.

The conventional breeding protocols designed for reducing phytic acid content, rely on the isolation of low phytic acid (*lpa*) mutations impairing the biosynthesis or the storage of phytic acid in the seed; the increased P and mineral cation bioavailability in the lpa mutant seeds so far isolated was confirmed by nutritional trials [9,10,11]. Low phytic acid mutations in maize can be classified into three categories: type 1 are mutations altering the biosynthetic pathway (MIPS, *myo*-inositol 3-phosphate synthase, catalyzing the step from glucose 6-P to myo-inositol[3]-monophosphate); type 2 are mutations altering the following phosphorylation steps (e.g., ITPK_S_, inositol tris/tetra kisphosphate kinases); type 3 are mutations altering the transport of phytic acid to the vacuole (MRP, multidrug-resistance-associated protein) [12,13]. In recent years, several breeding programs aimed at selecting new maize varieties with seeds characterized by a lower level of phytic acid compared to traditional cultivars, have been carried out [14,15]. The lpa phenotype has been isolated in different crops, maize [16,17,18], barley [19,20,21], wheat [22], rice [23,24,25,26] and common bean [27], by physical, chemical or transposon tagging mediated mutagenesis. In maize, there are three different *lpa* mutations (*lpa1*, *2* and *3*), with the *lpa1* mutation showing the lowest phytic acid content in the seed [16,17].

Due to the strong pleiotropic effects associated with the *lpa* mutations, the mutants thus far isolated are generally lethal; the challenge will be to isolate new lpa mutants in which the pleiotropic effects will be absent or dramatically reduced, allowing the *lpa* mutation to be viable.

*lpa1* mutation does not modify the total amount of seed P but reduces phytic acid content, thus leading to a proportionally increased level of free phosphate [18]. Owing to this, an HIP (high inorganic phosphate) phenotype is diagnostic for the presence of the *lpa1* mutation, making it quite easy to identify the lpa1 phenotype. The screenings so far used to identify the lpa mutants are destructive methods based on the quantification of P in the flour obtained by milling the seeds (as, for example, the Chen method [28]). For this reason, these screenings are not performed on the M1 generation but on the M2 progeny, with the consequent increase of time required to acquire the samples and number of samples to be analyzed.

Transposon tagging mutagenesis experiments [29] demonstrated that *lpa1* gene encodes a multidrug-associated-protein (MRP) named *ZmMRP4* (accession number EF586878). MRP proteins are transmembrane transporters involved in several functions such as organic ions transport, xenobiotic detoxification, oxidative stress tolerance and transpiration control [30,31]. 

The transposon tagging mutagenesis consists in introducing, by crossing, a known DNA sequence in the genome of a target species. If the insertion event occurs within a gene sequence, altering its expression, a mutant phenotype can be observed [32]. Because of the possibility of the transposon moving within the genome, transposon tagging mutagenesis provides the so-called mutable alleles that cannot be directly used in genetic improvement programs but that may be useful to clone the gene responsible for the mutated phenotype and to isolate stable excision events that lead to stable genomic mutations which can be used in genetic improvement programs.

The *Ac/Ds* transposon system of maize is often used in transposon tagging mutagenesis experiments. In fact, the elements of this transposon family can be transferred into the genome of different species, determining the generation of insertional mutants.

The *Ac/Ds* system is made up with two types of transposons: the autonomous element, which can transpose (the *Activator* element, *Ac*), and the non-autonomous element, which cannot transpose independently (the *Dissociator* element, *Ds*). Sequence analyses have shown that the *Ds* element is derived by deletion from the autonomous element of the family, with the loss of the function of one or more genes required for transposition. Therefore, only the *Ac* element encodes for the transposase, the enzyme required to mobilize both the *Ac* and the non-autonomous *Ds* elements [33]. Studies on *Ac/Ds* transposition have revealed a strong preference for insertion in regions of the genome in close genetic linkage to the donor site. In particular, it has been reported that the majority of *Ac* transpositions were within 10cM from the donor site [34]. This characteristic short-range transposition is used in regional mutagenesis studies to create multiple alleles in a target locus close to an *Ac* donor site.

In this work we present a new non-disrupting, fast and simple method to select lpa1 mutants. We describe the development and the screening of a transposon tagging mutagenized population which enabled the isolation of a new lpa1 mutant, named lpa1-5525.

## 2. Results

### 2.1. Transposon Tagging Mutagenesis

**Figure 1:**
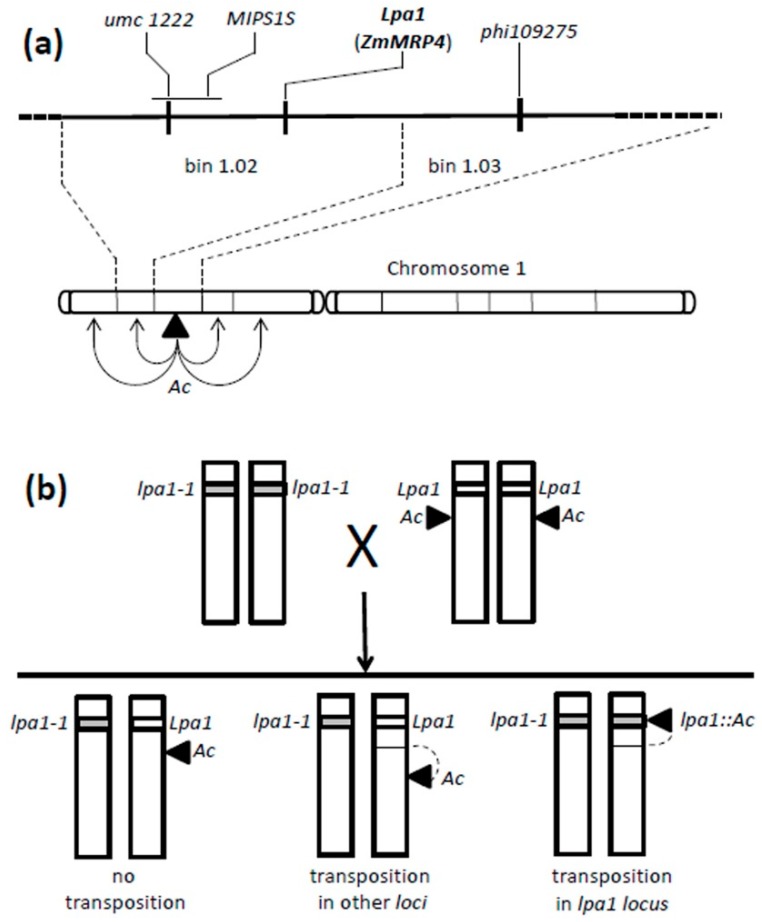
(**a**) Diagram of chromosome 1, the position of the *Ac* element and of *ZmMRP* locus, bin 1.02/1.03 are indicated. (**b**) Genetic scheme used to generate the F1 mutagenized population.

In order to isolate new lpa1 mutants, a transposon-mediated mutagenesis experiment was performed. The mutagenized population was generated by crossing the lpa1-1 mutant with a line carrying the *Ac* transposon (Figure 1). We chose an *Ac* line in which the transposon was inserted on the short arm of chromosome 1 in the 1.03/1.04 bin region, where *ZmMRP4* gene maps (Figure 1a).

We used *lpa1-1* homozygous plants as female and the *Ac* line as pollen donor. The mutagenized population, consisting of 4787 F1 seeds, was screened to find new lpa1 mutants.

### 2.2. Density Assay Screening of the Mutagenized Population

As previously reported, the *lpa1* mutation showed the lowest phytic acid content in the seed in comparison to the *lpa* mutations thus far characterized [16,17]. This mutation does not modify the total amount of seed P, but reduces phytic acid content, leading to a proportionally increased level of free phosphate [18]. The screening methods to identify *lpa1* mutations were thus far based on the identification of HIP (high inorganic phosphate) phenotypes by the quantification of the phosphate level in the seeds with disruptive methods [28].

**Figure 2:**
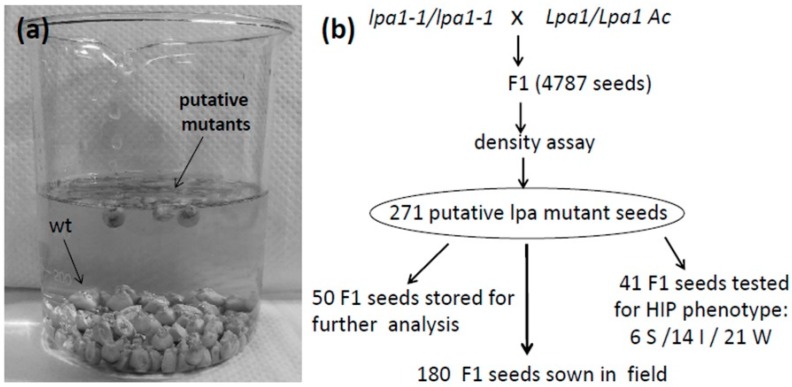
(**a**) Density assay used to isolate new lpa1 mutants: in the sucrose solution lpa1 mutant seeds float and wild type seeds stay on the bottom of the beaker. (**b**) Scheme of the experimental plan used to generate and to screen the F1 mutagenized population.

In order to isolate new lpa1 mutants inside the mutagenized populations, we developed a non-disrupting, fast, simple and cheap method. In particular, this assay was able to identify the mutants’ seeds because in an highly concentrated sugar solution (1.218–1.222 g/L) the lpa1 seeds, due to their lower density [35], can float, unlike the wild controls that sink and stay on the bottom of the beaker (Figure 2a). Among the 4787 F1 seeds screened with this test, 271 were identified as low density seeds (Table 1).

Out of these 271 seeds, 50 were put aside and stored for further analyses, 41 were tested for the HIP phenotype by the Chen method [28] and the remaining 180 were sown in the experimental field (Figure 2b).

### 2.3. Confirmation of the HIP Phenotype of the Low Density Seeds 

The density assay we developed was very rapid, cheap and easy, but it also revealed a limitation, i.e., since this screening is based only on the low density of the seeds to be selected, it did not allow the exclusive identification of lpa1 seeds, but it selected all seeds characterized by low density, including seeds affected by any kind of mutations impairing endosperm/embryo development and moldy seeds present inside the mutagenized population. For this reason, 41 out of the 271 seeds selected by the density assay were chosen and tested for the phosphate content (HIP phenotype) in order to verify that the low density character was associated with the lpa1 phenotype (Figure 2b). The determination of the free phosphorus content inside the seeds was carried out using a semi-quantitative colorimetric method based on the Chen reagent [28]. Based on the availability of the free phosphorus inside the seeds, it was possible to classify the seeds into four categories: WT (wild type—Free P < 0.3 mg/g), W (weak—0.3 mg/g < Free P < 0.5 mg/g), I (intermediate—0.5 mg/g < Free P < 1.4 mg/g) and S (strong—Free P > 1.4 mg/g) (Figure 4a). This assay allowed us to confirm the HIP phenotype for 20 out the 41 seeds selected with the density assay, with six seeds belonging to the Strong and 14 to the Intermediate categories (Figure 2b).

### 2.4. Molecular Analysis of the F1 Plants of the Mutagenized Population 

The lpa1-1 mutant was used as female parent in the initial cross we made to generate the mutagenized population. To identify and discard the contaminant seeds produced by the accidental self-fertilization of the lpa1-1 female parent, the 27 F1 plants, obtained from 180 F1 seeds sown in the open field (Figure 1), were genotyped by PCR analysis. The coding sequence of the *lpa1-1* allele is characterized, in comparison with the wild type allele, by the presence of a Single Nucleotide Polymorphism (SNP), C to T, in position 5759 with respect to the ATG on the genomic sequence. This allowed the design of two different forward primers, one specific for the wild type (ZmMRP430L) and the other specific for the *lpa1.1* allele (ZmMRP432L) [29,36]. We used the two specific forwards primers in combination with a reverse common primer (ZmMRP410R). Out of the 27 plants analyzed, 26 resulted in heterozygous *Lpa1/lpa1-1*; one plant was found to be a contaminant *lpa1-1/lpa1-1* and discarded (data not shown).

### 2.5. Screening and Selection of the Putative New lpa1 Mutant

**Figure 3:**
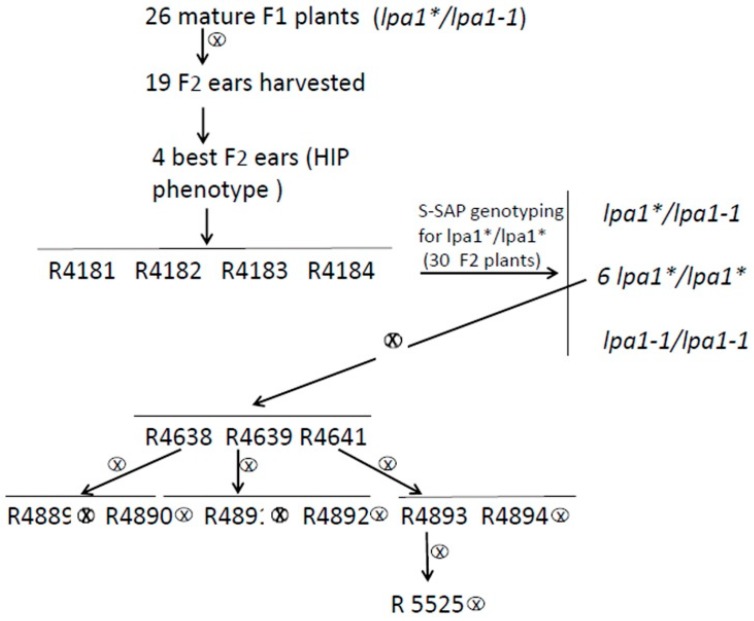
Scheme of the procedure used to obtain the NIL (Near Isogenic Line) lpa1-5525, homozygous for the new *lpa1* mutation.

Among the 26 F1 heterozygous plants (*Lpa1/lpa1-1*), we selected the 19 more vigorous that were self-fertilized (Figure 3). The four best F2 ears were tested for the HIP phenotype: 24 seeds were collected from each ear and the disruptive assay for free phosphate was performed. All the seeds tested were found to belong to the Strong or Intermediate categories, indicating an HIP phenotype for all the four selected ears and 50 seeds from each ear were sown in the field. 

Among the 90 plants that survived we selected the 30 more vigorous that were genotyped with the primers specific for *Lpa/lpa1* alleles, to identify the *Lpa1/Lpa1* F2 plants, i.e., the putative new lpa1 mutants, provisionally indicated as lpa1*.

The six F2 plants lpa1*/lpa1* were selfed and F3 seeds were tested for the HIP phenotype. The best F3 ears were selected (R4638, R4639, R4641) and generated the following six F4 families: R4889, R4890, R4891, R4892, R4893 and R4894. The F4 seeds were tested for HIP phenotype (Table 2) and the F4 ear R4893, showing high amount of S-I seeds and the best agronomic performance, was selected, sown in the field and the F4 plants were selfed. The F5 seeds (R5525) representing the lpa1* mutant were further analyzed.

### 2.6. Quantitative Analysis of Phosphorus Content

**Figure 4:**
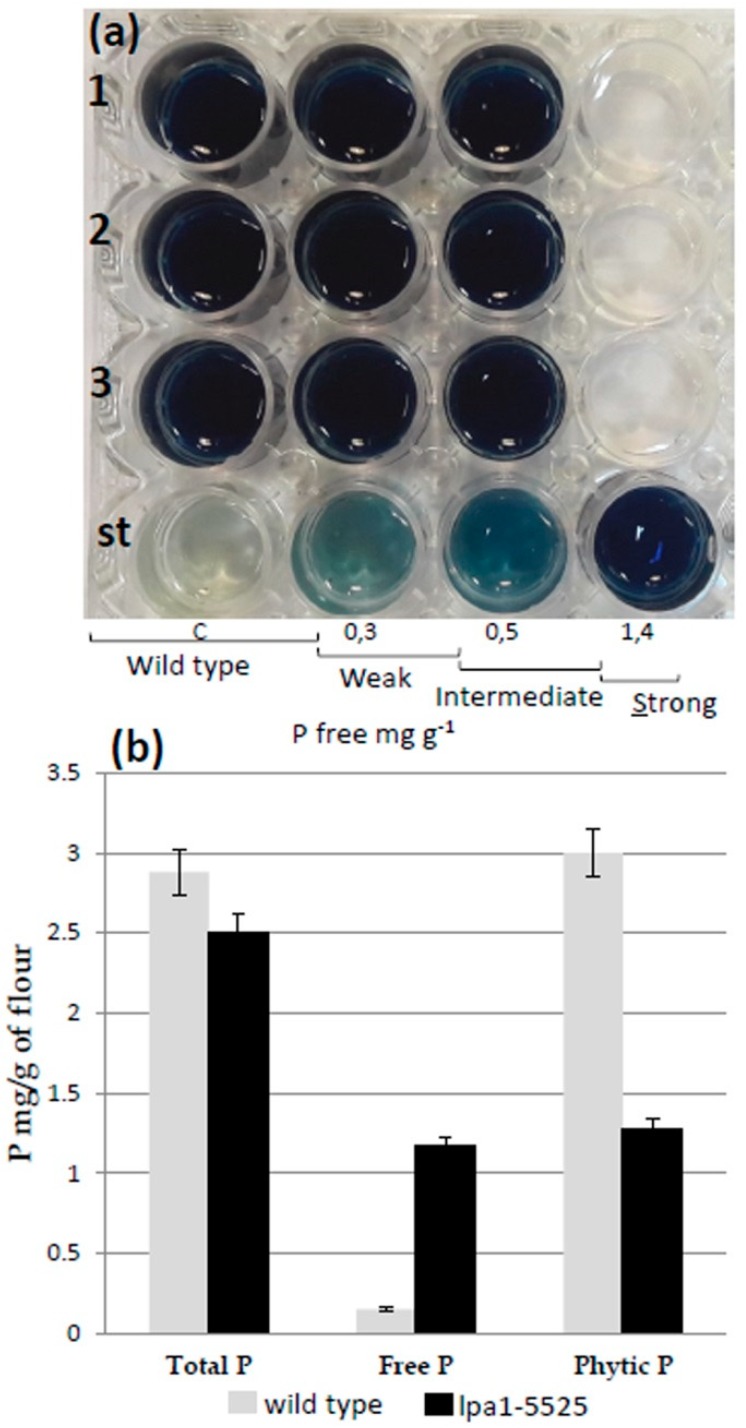
(**a**) Chen assay performed on F4 families mutant seeds. Microtiter rows 1, 2 and 3 show the results of the assay performed on nine mutant seeds, st row correspond to the standard used for the semiquantitative classification. (**b**) lpa1-5525 mutant and wild type control mature dry kernels were analyzed for total P, free inorganic P and phytic acid P amount. Values are expressed as milligrams of P (atomic weight 31) in 1 g of flour. Confidence intervals at 95% are shown.

The lpa1* mutant and wild type control seeds were tested for total P, free P and phytic acid content. No significant alterations in total P amount were observed between the putative new lpa1 mutant and the control: in fact, even if the new *lpa1* mutation caused approximately an eight-fold increase in the amount of free phosphate, this was balanced by a reduction of phytic acid that was nearly halved in comparison to the wild type content (1.28mg/g and 3mg/g, respectively) (Figure 4). In the lpa1 mutant, total phosphorus is 50% free P and 50% phytic P. 

### 2.7. Structure Analysis of the ZmMRP4 Locus

**Figure 5:**
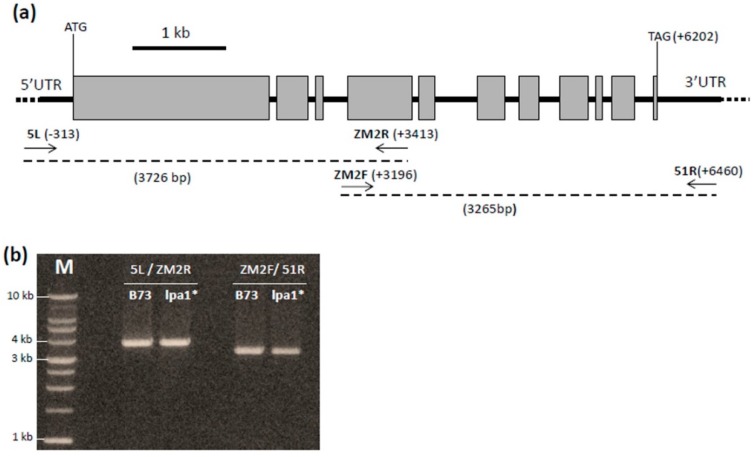
(**a**) Structure of the *ZmMRP4* gene. The primers used to amplify the coding sequence of *ZmMRP4* gene are indicated by the arrows. (**b**) Agarose gel showing the amplicons obtained amplifying wt (B73) and mutant (lpa1-5525) genomic DNA with primers 5L/ZM2r and ZM2F/51R.

The lpa1* mutant was isolated through a transposon-mediated mutagenesis experiment with the *Ac* element. To check for the presence of the 4.6 kb sequence of the *Ac* transposon into the *ZmMRP4* locus, the sequence of *lpa1* gene, spanning from the 5’UTR (nucleotide -313) to the 3’UTR (nucleotide 6460) (Figure 5a) was amplified in the lpa1* mutant and in wild type. The gel electrophoresis of the amplicons obtained failed to reveal any difference in length between wild type and the lpa1* mutant (also in the promoter region till about -1500bp, data not shown), thus suggesting the absence of the *Ac* transposon inside the *ZmMRP4* locus (Figure 5b).

## 3. Discussion

Phytic acid is an insoluble phosphate derivative, present in maize kernels, as in other seeds such as legumes. This element represents the main form of phosphorus reserve in the mature seed [3,37], degraded during germination by the activity of a group of enzymes called phytases [38]. Phytic acid has remarkable chelating properties and besides making phosphorus unavailable, it binds many minerals including calcium, iron, zinc, magnesium and manganese [10,39], which are not available for monogastric animals because of the lack of phytases in their digestive tract. According to the FAO data (www.FAO.org), maize is the leading cereal for production in the world, providing 1.04 billion tons of grain and occupying an area of 184.8 million hectares. For this reason, phytic acid is considered a strong worldwide food and feed antinutritional factor. 

Furthermore, in recent years, phosphorus has become one of the main sustainability issues because it is a non-renewable resource. Being widely used in agriculture as an essential component of fertilizers and feed, the increasing production of food has increased the rate of mobilization of reserves and the price of the mineral is continuously increasing. It is estimated that, at the current consumption rates, the phosphorus reserves available with the current technology will run out in 90 years. To date, several strategies have been developed to improve the bioavailability of phosphate in animal feed based on seeds and to reduce its environmental impact [40,41,42]. Some strategies were aimed at increasing phytase activity in the feed by incubation of the feed in water before administration, or by addition of purified phytases, other projects were based on the use of GMO maize that accumulates fungal phytases in kernels [43] or on the development of pigs engineered for the presence of phytases in the saliva [44]. Classical breeding programs, furthermore, allowed the isolation of mutant plants whose seeds were characterized by a low phytic acid content, the low phytic acid (lpa) mutants, (i.e., mutants defective in phytine synthesis). The peculiarity of these mutants concerns the distribution of phosphorus fractions: despite the fact that a smaller quantity of phytin is produced, phosphorus is accumulated in the seed during its maturation, remaining in a free form, directly assimilable by the monogastrics [16,17]. Thus far, various studies have reported the isolation of low phytic acid mutations in different species by the screening of a mutagenized population through Chen’s assay [12]. This disrupting assay is characterized by a high requirement of time and work [28]. In fact, F1 populations cannot be analyzed directly because of the disruption of the seeds analyzed, thus, we have to wait for F2 generation, and then for each F2 family, about 15–25 seeds have to be milled and the flour processed. For these reasons, to identify the putative mutagenized seeds in the F1 progeny, we developed and used a novel fast and cheap screening method based on the different density of lpa1 seeds in comparison with the wild type. In fact, in a previous paper [35], we showed that among the pleiotropic effects associated with the *lpa1* mutation there is also a significant reduction of seed density in comparison with the wild type. More recently, the role of the l*Lpa1* gene in influencing how densely starch granules are packed in the grain has also been confirmed in rice [45].

In this work, in order to isolate new mutations inside the *ZmMRP4/lpa1* locus, we generated a mutagenized population through a regional mutagenesis program based on the *Ac/Ds* transposons family. The initial crosses were performed between the *lpa1-1/lpa1-1* mutant line and a wild type line (*Lpa1/Lpa1*) carrying the *Ac* transposon [32] on the short arm of chromosome 1 (bin 1.02-03) where the *ZmMRP4* gene maps (Figure 1). This *Ac* line was chosen to increase the frequency of mutations at the locus *lpa1* because of the reported tendency of *Ac* transposons to reinsert in close linkage to the donor site [33]. The *lpa1-1* allele used in this work was characterized by a decrease in phytic acid synthesis (from 50% to 95%) and by a proportional increase of the level of free phosphorus inside the kernel. The *lpa-1*-*1* mutation leads to an alteration of the ABC transporter (ATP-Binding Cassette transporter) due to an SNP (Single Nucleotide Polymorphism) mutation, alanine to valine, on the amino acid 1432 [29]. This mutation impairs the transport and the subsequent impaired accumulation of phytic acid.

This density assay allowed the isolation of 271 low density putative lpa1 seeds among the 4787 F1 seeds tested (Figure 2 and Table 1). 

The selection for low density also allowed the selection of moldy seeds and seeds with other kinds of mutations impairing seed (embryo and/or endosperm) development. For this reason, as confirmation of the validity of this test for isolating lpa mutants, we checked for the free phosphate content a sub set of the 271 low density seeds and we identified 50% of seeds (20 out 40 tested) showing the HIP phenotype (Figure 2).

Furthermore, the low density assay did not allow the identification of the out of type lpa1.1 seeds coming from self-pollination events. We performed this analysis by genotyping the 27 F1 mature plants obtained from the 180 low density seeds sown in the open field and we found 26 plants heterozygous *Lpa1/lpa1* and one plant was an out of type homozygous *lpa1/lpa1,* which was discarded (data not shown). The best four F2 ears were further analyzed by Chen’s assay and sown in the open field, the six best F3 ears were analyzed by Chen’s assay and we selected the plant coded R 4892 that showed almost 100% of HIP seeds (Figure 3 and Table 2). After two more cycles of self-fertilization, F5 seeds were tested for the HIP phenotype, allowing the confirmation of the isolation of a new lpa1* mutant that we named lpa1-5525. Performing analysis regarding the P repartition (Total P, Free P and Phytic P) in this new mutant we registered values similar to those reported for lpa1-1 [16,36] (Figure 4b).

Molecular analysis excluded the presence of the *Ac* element in the *ZmMRP4* coding sequence due to the fact that the length of the two amplicons obtained by PCR is the same compared to the control (Figure 5). Of course, this result leaves open the possibility that the lpa1 mutant phenotype is caused by an *Ac* insertion into the promoter or in other regulative sequences, which could be very distant from the *lpa1* locus. However the stability of the lpa1-5525 progeny for what concerns the Free P content (in Figure 4a, nine out of 50 seeds were shown to be assayed for HIP) led us to suppose that we have isolated a “solid spontaneous mutation” in *ZmMRP4* locus because usually the insertion of an active *Ac* element produces “unstable alleles” [46]. For the same reason we could exclude an epigenetic origin for the lpa1 mutant here reported [47].

In conclusion, we developed a cheap, rapid and easy method to isolate lpa mutants. This method, not only allowed us to save money (fewer samples to be analyzed) and time (no need to wait for the F2 generation) but it also has a great potential based on the possibility to screen populations obtained with any methods of mutagenesis chosen (chemical, physical, transpositional). Furthermore, it is open to being adapted to the screening of low density seeds of other species than maize. Finally, we reported data about the isolation of a new lpa1 mutant, vital and thus useful in future breeding programs aimed at improving the nutritional value and decreasing the environmental problems associated with the high phytic acid content in maize seeds. Future work will be necessary to characterize the molecular lesion responsible for the lpa1 phenotype we isolated.

## 4. Materials and Methods

### 4.1. Genetic Stocks and Sampling Material

The lpa1-1 mutant stock was kindly provided by Dr. Victor Raboy, USDA ARS, Aberdeen, ID, USA. 

To generate the mutagenized population, we used a line (genetic background B73/W22) carrying the *Ac* transposon on short arm of chromosome 1 (bin 1.02/03) kindly provided by Dr. Thomas Brutnell, Shandong Agricultural University, China. 

### 4.2. Density Assay

Previous analyses [35] indicated that the lpa1 seeds were characterized by a lower density in comparison to the corresponding wild type controls. Starting from these data, we developed a non-disrupting, fast, cheap and rapid test to screen the mutagenized populations looking for the putative mutants containing the *Ac* transposon, *lpa1-1/lpa1-Ac*. 

The test was performed by putting the F1 seeds of the mutagenized populations in a concentrated sucrose solution, density 1.28 g/cm^3^ or 1,122g/cm^3^ at the temperature of 23 °C. This density assay allowed the isolation of the lpa1 putative mutants, which, because of their low density, were able to float while the wild type seeds stayed on the bottom of the beaker. 

This assay is non-disrupting, i.e., the selected seeds can be recovered from the beaker, rinsed, dried and used for further analysis, stored or sown. 

### 4.3. Assay for Free Phosphate Content in the Seeds

The Chen assay [28] was performed with some little modifications. The seeds were ground in a mortar with a steel pestle, and 100 mg of the flour obtained was extracted for 1 h at room temperature with 1 mL of 0.4 M HCl solution. After overnight incubation in a shaker at room temperature, 100 µL of extract was used for the free phosphate assay, adding 900 µL of Chen’s reagent (6 N H_2_SO_4_, 2.5% ammonium molybdate, 10% ascorbic acid, H_2_O [1:1:1:2, *v/v/v/v*]) in microtiter plates. After incubation of 1 h at room temperature the blue-colored phosphomolybdate complex was observed: the intensity of the blue color is directly proportional to the free phosphate content. The free phosphate content was quantified by using a spectrophotometer (λ 650 nm) and adopting a series of calibration standards obtained from a stock solution of KH_2_PO_4_.

### 4.4. Assay for Seed Phytate Content

In order to measure the content of phytic acid we used Megazyme’s kit K-PHYT 11/15 (Astori-Tecnica). Flour samples were obtained using a ball mill (Retsch MM200, Retsch GmbH Germany), grinding the seeds for 1 minute at 21 oscillations s^−1^ frequency. For each sample, in a beaker we added 20 mL of hydrochloric acid (0.66 M) to 1 g of flour that was vigorously stirred overnight at room temperature. 1 mL of extract was transferred into a 1.5 mL microfuge tube and centrifuged at 13,000 rpm for 10 min; then 0.5 mL of supernatant was transferred in a fresh microfuge tube and neutralized by the addition of 0.5 mL of sodium hydroxide solution (0.75 M). The neutralized extracts were submitted to enzymatic dephosphorylation reaction using the solutions supplied by the kit and trichloroacetic acid (50% *w/v*). The reactions were done in duplicate, to determine free phosphorus and total phosphorus. For colorimetric determination of phosphorus, 1 mL of sample extract was transferred into a 1.5 mL microfuge tube with 0.50 mL of color reagent (Ascorbic acid: 10%, Sulfuric acid: 1 M, Ammonium molybdate: 5%). The samples were mixed by vortex and incubated in a water bath at 40 °C for 1 h.

The standard phosphorus solutions were prepared as described in manufacturers’ instructions, with the only modification that after preparation the standards were not treated as samples, i.e., incubated at 40 °C, but were left at room temperature for 1 hour. The phytic acid content was quantified by using a spectrophotometer (λ 655 nm). The data obtained with the Megazyme software were expressed as g phytic acid/100 g of flour that we converted into mg phytic P/g of flour.

### 4.5. lpa1 Locus Molecular Genotyping

A molecular analysis was performed using *ZmMRP4* sequence-specific amplification polymorphism (S-SAP) markers that allowed the identification of the *lpa1-1* versus *Lpa1* allele. The allele-specific forward primers were designed on the single nucleotide substitution polymorphism in the *ZmMRP4* 10th exon [29,36]. The *Lpa1* wild type–specific forward primer was ZmMRP430L (5’-GTACTCGATGAGGCGACAGC-3’), whereas *lpa1-1* mutation-specific forward primer was ZmMRP432L (5’-GTACTCGATGAGGCGACAGTG-3’). The reverse primer ZmMRP410R (5’-CCTCTCTATATACAGCTCGAC-3’) was used to amplify both wild type and *lpa1-1* alleles.

The reaction mixture of the *Lpa1*/*lpa1-1* allele-specific amplifications contained an aliquot of genomic DNA, 1 × Green Go Taq buffer (Promega, Madison, WI), 2.5 µM MgCl2, 0.2 µM each of dATP, dCTP, dGTP, and dTTP, 0.3 µM of forward ZmMRP430L/ZmMRP432L-specific primer, 0.3 µM of reverse ZmMRP410R primer and 1.25 unit of Go Taq Flexy DNA polymerase (Promega), in a final volume of 25 µL. 

The reaction mix underwent an initial denaturation step at 94 °C for 2 min, 37 cycles of denaturation at 94 °C for 45 s, annealing at 62 °C for 1 min, extension at 72 °C for 1.5 min. Extension at 72 °C for 5 min was performed to complete the reaction. The *Lpa1* and *lpa1-1* amplicons were 468 bp long. The amplicons were loaded on 1% (*w/v*) agarose gels and visualized by ethidium bromide staining under ultraviolet light.

### 4.6. Structural Analysis of ZmMRP4 Locus in Putative New lpa 1 Mutant

*ZmMRP4* locus was amplified in wild type and in putative new lpa1 mutant using an high fidelity long range DNA polymerase (Platinum Super Fi DNA Polymerase, Invitrogen) with the primers 5L (5’-TGGTGAGGGGATCAGAGACG-3’) (forward primer, position -313) and ZM 2R (5’-GAACTTCCAAAGGCAAGGGACA-3’) (reverse primer, position +3413), ZM2F (5’-GGAAAAGTGAGCTCCAAAGTTTA-3’) (forward primer, position +3218) and 51R (5’-AAGCATCAGCTTCGGGTAATGT-3’) (reverse primer, position +6460). The primers positions are referred to the ATG on the genomic sequence.

The amplicons obtained were run on 1% agarose gel and visualized by ethidium bromide staining under UV light.

## Figures and Tables

**Table 1:** Density assay on mutagenized F1 seeds obtained by crossing *lpa1-1/lpa1-1* (codes R3962 to R3966 and R3893 and R864) with *Ac* line (R3967, R3970 and R916).

Code	Total Seeds Tested	Putative Mutant Seeds Isolated	Sucrose Solution Density (23 °C)
**R3962/R3967 (1)**	138	0	1.218
**R3962/R3967 (2)**	271	2	1.218
**R3962/R3967 (3)**	108	0	1.218
**R3962/R3967 (4)**	232	4	1.218
**R3962/R3967 (5)**	246	12	1.218
**R3962/R3967 (6)**	175	5	1.218
**R3962/R3967 (7)**	132	3	1.218
**R3963/R3967 (1)**	159	25	1.218
**R3963/R3967 (2)**	196	1	1.218
**R3963/R3967 (3)**	170	1	1.218
**R3963/R3967 (4)**	101	14	1.218
**R3963/R3970 (1)**	194	3	1.218
**R3963/R3970 (2)**	143	7	1.218
**R3964/R3967 (1)**	232	4	1.218
**R3964/R3967 (2)**	258	4	1.218
**R3964/R3967 (4)**	219	27	1.218
**R3964/R3967 (5)**	232	20	1.218
**R3964/R3967 (6)**	217	29	1.218
**R3965/R3970 (2)**	160	23	1.218
**R3966/R3970 (8)**	114	2	1.218
**R3893/R916-300**	166	8	1.218
**R864/R916/(3)**	335	20	1.222
**R864/R916 (5)**	285	14	1.222
**R864/R916 (8)**	159	27	1.222
**R864/R916 (9)**	145	16	1.222
**Total**	4787	271	

**Table 2:** Chen’s assay performed on F5 progenies. The S+I/total ratio is reported. S: Strong; I: Intermediate; W: Weak; WT: Wild Type.

Code	N° of Seeds Tested	Phenotype		S/I %
		S/I	W/WT	
**R4889** ⊗	24	13	11	54.17%
**R4890** ⊗	36	15	21	41.67%
**R4891** ⊗	36	8	28	22.22%
**R4892** ⊗	42	41	1	97.62%
**R4893** ⊗	18	17	1	94.44%
**R4894** ⊗	36	29	7	80.56%
**Total**	192	123	69	64.06%
